# Seasonal Dynamics of the Bacterial Community in Lake Urmia, a Hypersaline Ecosystem

**DOI:** 10.3390/biology14010075

**Published:** 2025-01-15

**Authors:** Robab Salami, Abbas Saidi, Mohammad Amin Hejazi, Bahman Panahi, Rasmieh Hamid

**Affiliations:** 1 Department of Cell & Molecular Biology, Faculty of Life Sciences & Biotechnology, Shahid Beheshti University, Tehran 1983969411, Iran; r.salami1990@gmail.com; 2 Department of Food Biotechnology, Branch for Northwest & West Region, Agricultural Biotechnology Research Institute of Iran (ABRII), Agricultural Research, Education and Extension Organization (AREEO), Tabriz 5156915-598, Iran; 3 Department of Genomics, Branch for Northwest & West Region, Agricultural Biotechnology Research Institute of Iran (ABRII), Agricultural Research, Education and Extension Organization (AREEO), Tabriz 5156915-598, Iran; panahi.lahroodi@gmail.com; 4 Department of Plant Breeding, Cotton Research Institute of Iran (CRII), Agricultural Research, Education and Extension Organization (AREEO), Gorgan 49166-85915, Iran; r.hamid@areeo.ac.ir

**Keywords:** beta diversity, hypersaline, 16S rRNA gene amplicon sequencing analysis, microbial diversity seasonal variations

## Abstract

Lake Urmia is one of the world’s most unique and hypersaline aquatic ecosystems. The aim of this study was to investigate the diversity, abundance and frequency of these microorganisms in water samples from the eastern regions of the lake over four seasons. Amplicon sequencing for the 16S rRNA gene was performed to examine bacterial communities in the samples. The study revealed significant seasonal variations in water quality parameters and their influence on the microbial communities. Majority and rarity analyses showed that winter and spring had higher core abundance and higher Gini index values, indicating a greater dominance of certain genera, while autumn and summer had a more balanced distribution. Analysis of beta diversity using the Bray–Curtis dissimilarity index emphasized that bacterial communities diverge most strongly in summer and winter, reflecting the significant changes in the environment with the season. Overall, understanding the seasonal variation in water chemistry and bacterial communities is critical for effective ecosystem management and conservation efforts.

## Simple Summary

Lake Urmia, one of the largest hypersaline ecosystems in the world, undergoes significant seasonal changes that affect its bacterial communities. This study investigated how microbial diversity varies over the seasons using advanced 16S rRNA gene sequencing. Water samples were collected in winter, spring, summer and autumn to investigate the effects of temperature, salinity and nutrient levels on bacterial diversity. The results showed that the bacterial communities differed most in summer and winter due to significant environmental shifts. Bacteria such as *Paucimonas* and *Salinibacter* dominated in certain seasons, reflecting their adaptation to the changing conditions. The study shows that winter harbors the greatest microbial richness, while summer has a lower diversity and favors salt-tolerant species. Understanding these seasonal patterns is critical to the management of Lake Urmia’s unique ecosystem and provides valuable insights for conservation efforts. These results also shed light on how microbial communities adapt to extreme conditions, which benefits studies on biodiversity and climate resilience.

## 1. Introduction

Saline habitats are found worldwide and serve as important reservoirs for novel microorganisms [1]. Among these habitats, hypersaline lakes stand out due to their extremely high NaCl concentrations and exceptionally dense microbial populations [2]. These environments exhibit seasonal salinity gradients, mainly caused by water evaporation, which in turn influence the structure and composition of microbial communities and allow the spread of highly specialized organisms [3].

There are a variety of hypersaline environments in Iran, including salt mines, salty deserts, salt rivers and especially hypersaline lakes. Lake Urmia in north-western Iran is one of the most biodiverse habitats in the world and harbors a rich variety of flora and fauna, including a considerable reservoir of moderately halophilic and halotolerant microorganisms [4]. Halotolerant microorganisms do not require high salinities but thrive at concentrations of 100–200 mM NaCl, while halophiles that require higher concentrations are categorized as light, medium and extreme halophiles [5]. These halophiles are found in all three domains of life—Archaea, Bacteria and Eukarya. Halophilic bacteria can thrive in a wide range of salt concentrations (3–15% NaCl *w*/*v* and beyond), whereas truly halophilic archaea are restricted to high-salinity environments [6]. The adaptation of bacteria and archaea to salt marshes is facilitated by the high osmotic pressure, the accumulation of organic solutes in their cytoplasm and their ability to maintain osmotic equilibrium [7].

Aquatic bacteria play a crucial role in the maintenance of natural aquatic ecosystems by participating in essential biogeochemical processes such as nutrient cycling, respiration and the removal of pollutants [8]. Six arsenic-resistant bacterial strains belonging to the genera *Shouchella*, *Salipaludibacillus* and *Evansella* were identified from Lake Urmia. These strains exhibited high tolerance values and possessed important detoxification genes such as *arsC*. Arsenic contamination poses a significant threat to the environment and health, but these bacteria offer the potential for a two-step bioremediation process to effectively combat arsenic pollution [9]. Research shows that environmental factors significantly influence the structure of bacterial populations in different seasons. Understanding the seasonal variations in microbial diversity and distribution is critical to the effective utilization of these microbiomes in various applications [10]. For example, studies conducted in river catchments in North Carolina have shown that bacterial communities shift seasonally, with gamma, alpha and beta proteobacteria predominating, while actinobacteria are more abundant in dry seasons [11]. Similarly, seasonal changes in Funtana Bay, in the northern Adriatic Sea, showed fluctuations in cyanobacteria populations, with other high-ranking species present throughout the year [12].

Culture-independent studies of hypersaline environments worldwide have revealed unexpectedly high microbial diversity and numerous uncharacterized halophilic microbes [13]. Next-generation sequencing (NGS) technologies such as Illumina MiSeq have significantly improved our ability to analyze microbial communities at high resolution and throughput [14].

Hypersaline ecosystems, including various terrestrial lakes and deep-sea basins with salt concentrations three times that of seawater, are often characterized by additional extreme conditions such as high alkalinity, low oxygen levels and high UV irradiation [15]. With its remarkable microbial diversity, Lake Urmia offers unique opportunities to study the characteristics and ecological significance of moderately halophilic and halotolerant bacteria.

## 2. Materials and Methods

### 2.1. Sampling Location

**Figure 1:**
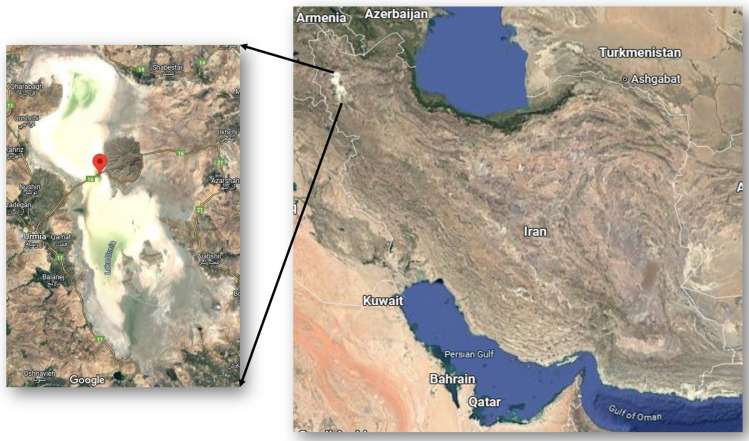
Location of Lake Urmia in Iran, located on the left side of the map. The map was created using the online tool Google Maps.

Lake Urmia is located between the provinces of East Azerbaijan and West Azerbaijan in Iran, to the west of the southern part of the Caspian Sea. The coordinates for Lake Urmia are 37°47′45.0168″ N 45°22′51.1212″ E (Figure 1). The water samples from the lake were collected in 2021 during four different seasons: in February, May, August and November. Each sample contained a total of 2 L of water taken from the lake at a depth of 10 cm. The samples were collected in sterile containers and transported to the laboratory. During transport, the samples were kept at a temperature of 4 °C and refrigerated until further analysis.

### 2.2. Characterization of Physico-Chemical Properties

Electrical conductivity (EC), salt concentration and pH were measured using EC meters (Cond 315i/SEC, Wissenschaftlich-Technische Werkstätten GmbH, Germany), following the manufacturer’s instructions. For the analysis and calculation of nitrate, potassium and sodium concentrations, the samples were analyzed with a flame photometer (Lambda 35). In addition, other physico-chemical properties, including total dissolved solids (TDS), calcium, magnesium, chloride and sulfate, were determined using atomic absorption spectroscopy. All measurements were conducted according to the guidelines of the American Public Health Association (APHA).

### 2.3. Environmental DNA Extraction

Debris and eukaryotic cells were first removed using glass microfiber filters (Whatman GF/D). To prepare the samples for 16S rRNA gene amplicon sequencing, a pre-filtration step was performed using 8 μm filters (round filter, Filttrak) to collect biomass from the samples collected during the four seasons. The samples were then further filtered through 0.22 μm filters (Schleicher and Schuell) to effectively remove larger particles and microalgae contaminants. A modified protocol based on Benlloch et al. (2001) was used for the extraction of environmental DNA [16]. The filters were cut into small pieces with a sterile razor blade, vortexed in 2 mL of sterile water and transferred to separate Falcon tubes. To this solution, 200 μL of 10% SDS was added. The mixture was then treated with proteinase K and incubated at 55 °C for 120 min, followed by a short incubation at 100 °C for 2 min. The lysates were then subjected to chloroform–isoamyl alcohol extraction (in equal volumes), and the supernatants were transferred to new sterile tubes. Subsequently, 1 cc of cold isopropanol was added to the solutions and incubated at room temperature for 24 h. After incubation, the solutions were centrifuged at 10,000× *g* rpm for 10 min. The resulting pellets were washed with 70% ethanol and dried at room temperature (22 °C). The quantity and quality of extracted DNA was determined using 0.7% agarose gel electrophoresis and a NanoDrop 1000 Thermo Scientific spectrophotometer, respectively.

### 2.4. 16S rRNA Gene Amplification and Sequencing

The purified environmental DNA was sequenced using the Illumina MiSeq platform, with a maximum read length of 300 base pairs and 30% Phi X to improve diversity and error correction. More than 100,000 reads per sample provide ample data for in-depth community analysis. Sequencing was performed at Macrogen Europe BV and Psomagen Inc. (Seoul, Republic of Korea) using a paired-end library method. We performed some 16S rRNA gene amplicon sequencing with primers used for sequencing the variable regions V3 and V4 of the 16S rRNA gene according to Illumina. The library was created with the primers Bakt_341F (CCTACGGGG-NGGCWGCAG) and Bakt_805R (GACTACHVGGGGTATCTAATCC). The primers Bakt_341F and Bakt_805R were specifically designed for the amplification of the V3 and V4 variable regions, which are commonly analyzed in microbial ecology studies to gain insights into the diversity and composition of bacterial populations [17].

### 2.5. Preprocessing and Taxonomical Classification

The raw 16S rRNA gene amplicon sequencing data were quality-checked with FastQC v.0.12.1 and trimmed with Trimmomatic v0.39. For taxonomic profiling of the mi-crobial communities, the R package (R ver. 4.4.1) was used to classify the 16S taxonomy, specifically the microclass pipeline [18]. For taxonomic classification, the R function taxMachine was used, which provides classification information based on posterior probabilities from a multinomial model.

The K-mer counting approach in this pipeline discards sequences with ambiguous features to allow a clearer taxonomic assignment. The d-score was calculated to assess classification confidence, with a d-score close to 0 indicating a higher probability of misclassification near a decision boundary. The r-score and associated probability were used to filter out unusual sequences [18].

### 2.6. Diversity and Core Microbiome Analysis

We applied a negative binomial distribution to normalize our samples, following the method recommended by McMurdie and Holmes, (2013) and implemented in the Bioconductor package DESeq2 [19,20]. This approach effectively accounts for differences in library size and biological variability. After normalization, we compared the taxonomic profiles of phyla, class, family and genus obtained from 16S rRNA gene amplicon sequencing. In addition, we compared bacterial abundances and diversity indices from 16S rRNA gene amplicon sequencing data with those obtained from metataxonomic 16S rRNA gene sequencing data.

To assess taxonomic alpha diversity, we used the Chao1 richness estimator together with Shannon, Simpson and inverse Simpson indices. These indices provide information on the richness and evenness of the microbial communities. The dominance of the bacterial community was measured using the dominant function from the microbiome R package, while the core microbiome was determined across different samples using the core abundance function. This function in the microbiome R package calculates the core abundance of the community index. This index indicates the proportion of the community made up by the core taxa.

To analyze beta diversity, we used the Bray–Curtis distance, a non-phylogenetic metric suitable for sparse count data. Calculations were performed using the distance function in the R package phyloseq. To visualize the taxonomic dissimilarity between samples, we used principal coordinate analysis (PCoA). We also used heat maps to visualize seasonal variations in the microbiome and performed a Venn analysis to identify patterns and overlaps. Principal component analysis (PCA) of physico-chemical properties and bacterial dynamics was performed using GenStat 12th Edition.

## 3. Results and Discussion

### 3.1. Physico-Chemical Properties of Lake Urmia

Seasonal fluctuations in water quality parameters such as temperature, salinity, pH and nutrient concentrations have a significant impact on microbial communities [21]. Experiments and data analyses provide valuable insights into how these conditions influence microbial diversity, abundance and community structure throughout the year. Understanding these seasonal variations is essential for the optimization of sampling strategies and the accurate interpretation of 16S rRNA gene amplicon sequencing data, as they can influence microbial distribution and activity [22]. In addition, the physico-chemical properties of water, including salinity and nutrient content, directly affect microbial metabolic processes and community dynamics. The study of these factors establishes correlations between environmental conditions, microbial functions and interactions and provides insight into how microbial communities adapt to change [23].

Table 1 shows the physico-chemical properties of Lake Urmia, focusing on the seasonal fluctuations of the water quality parameters. Electrical conductivity (EC) values were significantly higher in autumn and summer, probably due to increased evaporation. In contrast, winter and spring had lower EC values, probably due to dilution by precipitation and runoff, which reduced the concentration of dissolved salts. Fluctuating salinity levels, as indicated by the high EC values in autumn and summer and the lower values in winter and spring, can affect aquatic organisms, especially those sensitive to salinity changes [24]. Species that are adapted to brackish conditions may thrive during periods of higher salinity, while others may struggle, potentially leading to shifts in species composition or upsetting the ecological balance [25].

The results showed that pH values were significant in winter and spring. A higher pH during these seasons could be due to a lower input of organic acids or an increased buffering capacity during these periods [26]. In contrast, the lower pH in autumn and summer could be due to seasonal changes in biological activity or acidification processes. As shown in Table 1, total dissolved solids (TDS) decreased from autumn to spring, probably due to dilution from increased runoff or precipitation. However, TDS increased in summer and autumn, indicating an accumulation of dissolved solids, possibly owing to the decrease in water level due to evaporation [27].

Magnesium levels were significantly higher in autumn and summer than in other seasons, suggesting that seasonal evaporation may concentrate magnesium in the lake. Lower values in winter could reflect greater dilution or lower magnesium sources during the colder months. Potassium and sulfate levels followed a similar seasonal pattern, with higher levels in autumn and summer and lower levels in winter and spring. Fluctuations in nutrients such as nitrate, phosphate and sulfate probably influence the primary productivity of the lake [28].

The ionic composition of Lake Urmia consists mainly of six major ions: Cl^−^, Na^+^, SO_4_^2−^, Mg^2+^, K^+^ and Ca^2+^, which together account for more than 99.9% of the total ions. This composition indicates that evaporation, together with the precipitation of calcium sulfate and calcium carbonate, contributes significantly to the salinity, electrical conductivity (EC), total dissolved solids (TDS), hardness and alkalinity of the lake [29]. Studies at Lake Urmia have documented extremely high chloride concentrations, primarily due to high evaporation and low water levels. In line with our findings, previous studies have reported chloride concentrations in Lake Urmia ranging between 176.2 and 201.3 g/L [5,30].

The results also showed that nitrate and phosphate levels were significantly elevated in autumn and summer, which can lead to algal blooms and a deterioration in water quality. Conversely, lower nutrient levels in winter and spring can reduce the risk of such blooms but also affect primary production rates. Overall, the data show considerable variability in the water chemistry of Lake Urmia throughout the year. A high diversity value was found in summer, which is considered a dry month and is characterized by low water levels due to evaporation, resulting in concentrated amounts of nutrients, especially Ni rates, during these seasons [31]. These fluctuations are determined by factors such as precipitation, evaporation, runoff and biological activity, which together determine the water quality and chemistry of the lake. Understanding these seasonal trends is critical to the effective management and maintenance of the lake’s ecosystem.

### 3.2. Microbiome Analysis

Characterizing the microbiome over different seasons improves our understanding of the functional role of microorganisms in the biogeochemical cycles of the lake. These microorganisms are crucial for nutrient cycling, organic matter decomposition and other ecological processes, so their seasonal behavior is critical to the health of the lake ecosystem. Conducting a seasonal microbiome analysis of Lake Urmia is essential to understand the effects of environmental fluctuations on microbial communities, predict future changes and take targeted conservation measures [32]. This analysis provides valuable insights into microbial ecology in extreme environments and supports efforts to sustainably manage one of the most diverse hypersaline aquatic ecosystems in the world. Below, we present a detailed analysis of the metagenome-based microbial diversity of Lake Urmia across seasonal fluctuations. This study investigates how microbial communities respond to changing environmental conditions throughout the year and provides insights into the interactions between microorganisms and their hypersaline environment.

### 3.3. Coverage Analysis

Coverage analysis enables better comparisons between different samples or studies. It provides a consistent representation of data across samples, enabling meaningful comparisons and reliable conclusions about similarities and differences between microbial communities [33]. The coverage index estimates the minimum number of samples required to cover a certain proportion (50% by default) of the total bacterial community. Our metagenome analysis showed that just two samples per season was sufficient to cover 50% of the observed genera. This suggests that the current sampling adequately captures the genus composition of the bacterial communities in the different seasons.

### 3.4. Taxonomic Profiling Based on 16S rRNA Gene Sequences

**Figure 2:**
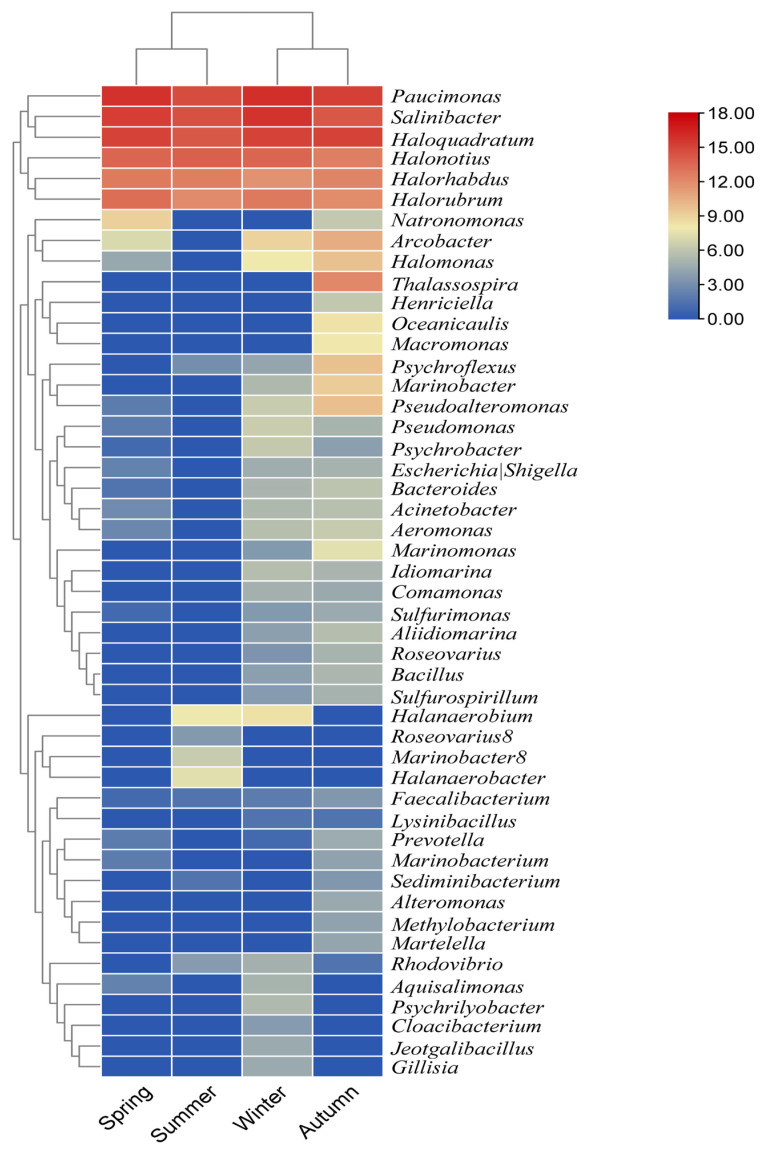
Relative abundance of different bacterial genera in each of the four seasons—winter, spring, summer and autumn—in Lake Urmia.

Figure 2 shows the relative abundance of the different bacterial genera in autumn, winter, spring and summer. These results illustrate the fluctuations in bacterial populations throughout the year, with certain genera dominating in certain seasons and others being less abundant. In autumn, winter and spring, *Paucimonas* dominates with an abundance of 32.25%, 35.83% and 35.88%, respectively. This shows that *Paucimonas* thrives under the conditions typical of these seasons. *Paucimonas* activity is crucial for maintaining the productivity and health of the lake ecosystem, especially under oligotrophic (nutrient-poor) conditions where microbial decomposition is critical for nutrient availability [34]. Our results are consistent with previous reports and confirm the role of *Paucimonas* in decomposition and nutrient cycling processes during these seasons.

The dominance of *Salinibacter* in summer with an abundance of 24.41% indicates a significant change in environmental conditions that favors this halophilic (salt-loving) bacterium [35]. *Salinibacter* is known to thrive in high-salinity environments and contributes to sulfur and nitrogen cycling and the degradation of organic compounds [36]. Its strong presence in winter and summer suggests that it plays a central role in nutrient cycling under changing salinity conditions influenced by seasonal evaporation and precipitation. Metagenome studies have also identified *Haloquadratum walsbyi* as the most abundant archaeon in saline environments [37].

Genera such as *Halonotius* and *Haloquadratum*, which occur across multiple seasons but at relatively low abundance, indicate a broad ecological range but lower competitiveness compared to the dominant genera. Metagenomic analysis of water samples from six sites in Lake Urmia revealed microbial communities predominantly associated with *Haloquadratum* (59.3%) and *Halonotius* (9.1%). Similar to other hypersaline lakes, the bacterial community in Lake Urmia is largely dominated by *Salinibacter ruber* (23.3%) [29]. Both *Halonotius* and *Haloquadratum* play a crucial role in hypersaline lake ecosystems. Their metabolic processes, including the degradation of complex organic materials, contribute to carbon and nitrogen recycling, stabilize the structure of the microbial community and support ecosystem functionality [13]. Their metabolic processes facilitate the recycling of carbon and nitrogen, which are essential for the lake’s food web. Their presence contributes to the stability of the microbial community structure and supports the overall functioning of the salt lake ecosystem.

Other bacterial genera found in Lake Urmia are *Halorhabdus*, *Faecalibacterium*, *Psychrobacter* and *Halomonas*, as shown in Figure 2. *Faecalibacterium*, for example, is associated with the breakdown of complex carbohydrates and the production of butyrate, a vital short-chain fatty acid [38]. The presence of these bacteria in low abundance during certain seasons suggests that they may play a special role under certain environmental conditions, such as lower temperatures or limited nutrient availability. Their activities increase the microbial diversity and functional resilience of the lake ecosystem during these periods.

### 3.5. Distribution of Bacterial Genera

**Figure 3:**
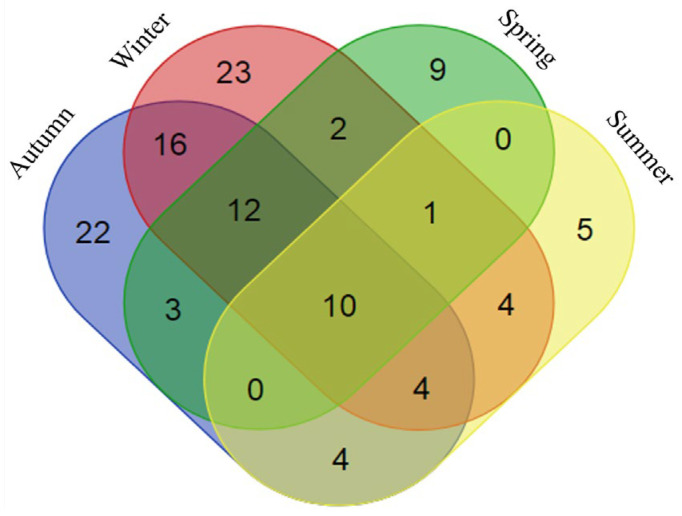
Seasonal distribution of the annotated bacterial genera over the four seasons (autumn, winter, spring and summer) in Lake Urmia.

Our analysis of the seasonal variation of bacterial communities in Lake Urmia provides insights into the distribution and diversity of bacterial genera throughout the year. This study investigated the presence of annotated bacterial genera during each of the four seasons (autumn, winter, spring and summer) and identified important patterns in bacterial diversity and community structure (Figure 3).

Several bacterial genera were found across all seasons, including *Paucimonas*, *Halorhabdus*, *Halonotius*, *Haloquadratum*, *Prevotella*, *Salinibacter*, *Faecalibacterium*, *Halomonas* and *Halorubrum*. The year-round occurrence of these genera indicates their ability to adapt to the changing environmental conditions in Lake Urmia. These genera may possess traits that allow them to thrive in different seasons, demonstrating ecological resilience and versatility [39].

In contrast, some genera showed a season-specific distribution, indicating adaptation to certain environmental conditions. For example, *Sulfophobococcus* was found exclusively in samples collected during winter, spring and summer, suggesting its preference for the conditions in these seasons or its specific adaptability to them. This distribution could be influenced by seasonal changes in temperature, salinity or nutrient availability that promote the growth of *Sulfophobococcus* at particular times of the year [40]. In addition, unique genera were observed in each season: Acetobacter and *Methylobacterium* in autumn, *Planococcus* and *Thiobacillus* in winter, *Pseudolabrys* and *Pyrococcus* in spring, and *Dorea* and *Halanaerobacter* in summer. These seasonal genera illustrate distinct ecological niches and environmental conditions shaping the bacterial community composition. The occurrence of unique genera in different seasons suggests significant shifts in microbial communities in response to changing environmental factors such as temperature, pH and nutrient levels [41]. These findings emphasize the importance of considering seasonal fluctuations when studying microbial communities. The persistent presence of certain genera throughout the year highlights their adaptability to varied conditions, whereas the selective presence of others during specific seasons underscores the influence of environmental changes on bacterial diversity. Understanding these patterns provides valuable insights into microbial adaptation, ecological interactions and the potential impact of environmental changes on bacterial communities.

This knowledge is crucial for applications in agriculture, environmental management and microbial ecosystem studies [42]. A phylogenetic analysis based on 16S rRNA gene sequences of culturable halophilic bacteria in Lake Urmia revealed that the isolated bacteria belonged to two main taxa: Gammaproteobacteria (including Pseudomonas, *Marinobacter*, *Idiomarina* and *Halomonas*) and Firmicutes (including *Bacillus* and *Halobacillus*). Most isolates were also capable of nitrate reduction, suggesting their involvement in nitrogen cycling processes within the lake [43]. Moderately halophilic bacteria such as *Marinobacter*, *Idiomarina* and *Halomonas* strains have been reported to degrade organic pollutants and organic nitrogen compounds found in food, organic materials, fertilizers, toxins and explosives [44].

### 3.6. Alpha Diversity

Our study investigated bacterial diversity in Lake Urmia across four seasons, and the results are summarized in Table 2. The data reveal interesting patterns in genus richness and diversity indices. Winter exhibited the highest genus richness, with 72 bacterial genera observed, followed closely by autumn with 71 genera. In contrast, spring showed a marked decline to 37 genera, while summer recorded the lowest richness at just 26 genera. The Shannon index, which considers both the frequency and evenness of genera, indicated that autumn had the highest diversity (1.835828), suggesting a well-balanced and evenly distributed bacterial community compared to the other seasons. This finding supports the hypothesis that temperate conditions in autumn provide stable habitats and resources, fostering microbial diversity [1]. On the other hand, winter recorded a lower Shannon index (1.516104), implying reduced evenness and diversity despite high genus richness. This could be attributed to colder temperatures and potentially lower metabolic rates, which may limit the growth and even distribution of genera [45].

The Simpson index, which measures the likelihood that two randomly selected individuals from a sample belong to the same genus, highlighted that summer had the highest index value (0.7877350), indicating the lowest diversity. This suggests that a few genera dominate under extreme conditions in summer, consistent with previous observations in hypersaline environments, where high salinity selects for specialized taxa [2,46]. Autumn and spring exhibited intermediate Simpson’s index values (0.7748228 and 0.7538326, respectively), while winter had the lowest Simpson’s index (0.7320779), reflecting the highest diversity according to this measure.

The inverse Simpson’s index, which inversely correlates with the likelihood that two randomly selected individuals belong to the same genus, revealed that summer had the highest value (4.711091). This suggests that, despite low genus richness, certain highly adapted genera dominate. Autumn, spring and winter followed with values of 4.440946, 4.062276 and 3.732428, respectively, reflecting seasonal differences in community structure and diversity.

### 3.7. Dominance and Rarity Analysis

Dominance and rarity analyses are essential for understanding microbial community structures and their ecological dynamics. These analyses provide insights into the distribution of microbial taxa, their relative abundance and the overall balance within the community. Table 3 highlights various indices characterizing the bacterial communities in Lake Urmia across the different seasons, offering insights into dominance, diversity and core abundance. The values for dominance by proportion (dbp) indicate a pronounced prevalence of certain genera in spring (0.359) and winter (0.358). This suggests that these genera exhibit ecological advantages or adaptations that enable them to thrive during these seasons, likely influenced by favorable environmental conditions or competitive interactions [47]. In contrast, summer showed a lower dbp value (0.288), indicating a more even distribution of genera and less dominance of a single genus. This shift may reflect increased competition or changes in environmental conditions that promote a more balanced community structure during the warmer months [48].

The observed fluctuations in absolute and relative bacterial abundance further emphasize these seasonal shifts. Absolute abundance was highest in winter (65,596) and lowest in summer (27,542). This pattern is consistent with findings from similar studies, which show that bacterial abundance varies seasonally due to changes in factors such as temperature and nutrient availability [49]. Relative abundance followed the same trend, with winter showing the highest value (0.358) and summer the lowest (0.288). This decrease in abundance during summer may suggest a reduction in bacterial taxa or a broader diversification of the community, possibly in response to seasonal environmental changes [50].

The Simpson dominance index and Gini coefficient provide additional insights into community structure. The Simpson index increased slightly from autumn (0.225) to summer (0.212), indicating a trend towards a more even distribution of genera, though dominance of specific taxa has decreased (Simpson, 1949). The Gini coefficient, ranging from 0 (complete equality) to 1 (complete dominance), also increased slightly from autumn (0.908) to summer (0.994). These results suggest a more even distribution of bacterial genera across the seasons. However, the subtle rise in dominance indices from autumn to summer reflects nuanced changes in community composition [51].

The analysis of rare and uncommon genera, indicated by the log-modulo skewness, revealed similar values across all seasons (approximately 2.05), with a slight decrease from autumn to summer. This trend suggests a more balanced distribution of rare and uncommon genera during summer, possibly driven by shifts in environmental conditions or community interactions that promote an even bacterial distribution [52]. The decrease in both absolute and relative abundance in summer supports the idea of a broader diversification of bacterial taxa or a response to seasonal stressors as observed in other hypersaline environments [53].

Core abundance assesses the stability and persistence of dominant microbial taxa within a community (Table 4). The results indicate that core abundance was highest in spring (0.995) and summer (0.994), suggesting that these seasons provide conditions conducive to a stable core microbiome (Table 3). This stability may stem from favorable environmental conditions that support consistent growth of key taxa. Seasonal stability in core microbiomes is often observed in aquatic environments, where certain bacterial taxa persist due to relatively stable conditions [54]. For example, the presence of a stable core microbiome during favorable seasons may indicate periods of abundant nutrients or optimal temperatures that favor the growth of important microbial genera [55]. Conversely, autumn exhibited lower core abundance (0.908), indicating greater variability in the core microbiome. This variability may result from transient environmental conditions, as the lake undergoes changes in temperature, nutrient availability and other ecological factors [56]. Such fluctuations are known to affect microbial community structure and lead to shifts in key taxa [57].

The Gini index measures the unequal distribution of genera within a community. Higher values indicate dominance of a few taxa, while lower values reflect a more balanced distribution. In this study, winter (0.968) and spring (0.967) showed the highest Gini index values, suggesting strong dominance of specific bacterial genera during these seasons. This pattern may result from stable environmental conditions that favor growth of dominant taxa [58]. The stronger dominance in winter and spring could also be related to less competition and fewer environmental stressors, allowing dominant taxa to establish a stronger presence [59]. Conversely, autumn (0.957) and summer (0.963) showed lower Gini index values, indicating a more balanced distribution of bacterial taxa. This trend towards balance in autumn and summer may arise from heightened competition or changing conditions that encourage a more diverse microbial community [60].

### 3.8. Beta Diversity Analysis

After analyzing alpha diversity and the dominance of bacterial communities in the different seasons of the lake, the differences in the composition of genera between these communities were assessed using beta diversity analysis. Beta diversity measures the variation in genus composition between samples or ecosystems (Table 5).

**Figure 4:**
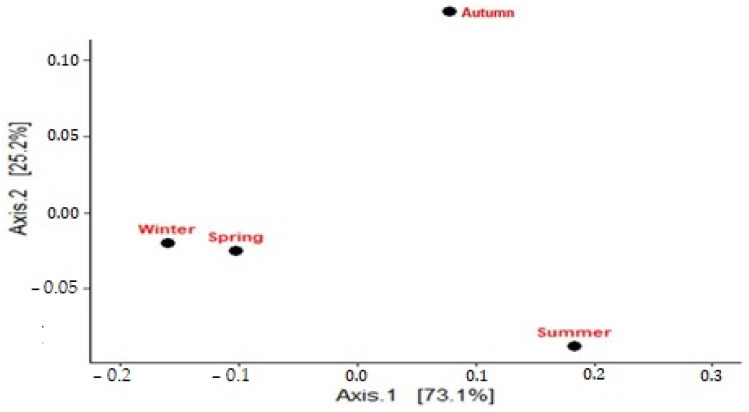
Principal coordinate analysis (PCA) of bacterial communities across seasons in Lake Urmia: distribution of bacterial communities based on the Bray–Curtis dissimilarity index, with samples from winter, spring, summer and autumn.

The Bray–Curtis dissimilarity index was used to quantify the differences in bacterial community composition between seasonal samples. The highest dissimilarity (0.3505) was observed between winter and summer samples, suggesting that these seasons harbor the most distinct bacterial communities. This significant dissimilarity can be attributed to significant changes in environmental conditions between winter and summer, such as significant temperature fluctuations, variations in nutrient availability and changes in water chemistry. For example, temperature plays a crucial role in the composition of microbial communities by influencing metabolic rates and community structure [61]. Similarly, the availability of nutrients can influence community dynamics by favoring certain taxa under different seasonal conditions [62]. These factors likely contribute to the observed shifts in bacterial communities between winter and summer. The lower dissimilarity between winter and spring samples (0.0828) suggests that the bacterial communities are more similar to each other in these seasons. This high similarity suggests that environmental conditions or resource availability gradually transition from winter to spring, resulting in a more consistent microbial community structure. This result is consistent with other studies indicating that transitional periods between seasons often lead to gradual changes in microbial communities rather than abrupt shifts [63]. The PCoA plot (Figure 4) illustrates the separation of bacterial communities along the first principal coordinate (PC1), with winter and spring samples showing some separation from the other seasons. This plot confirms the Bray–Curtis dissimilarity results and highlights winter as a particular period in terms of bacterial community composition.

### 3.9. Principle Component Analysis of Physico-Chemical Properties and Bacterial Dynamics

**Figure 5:**
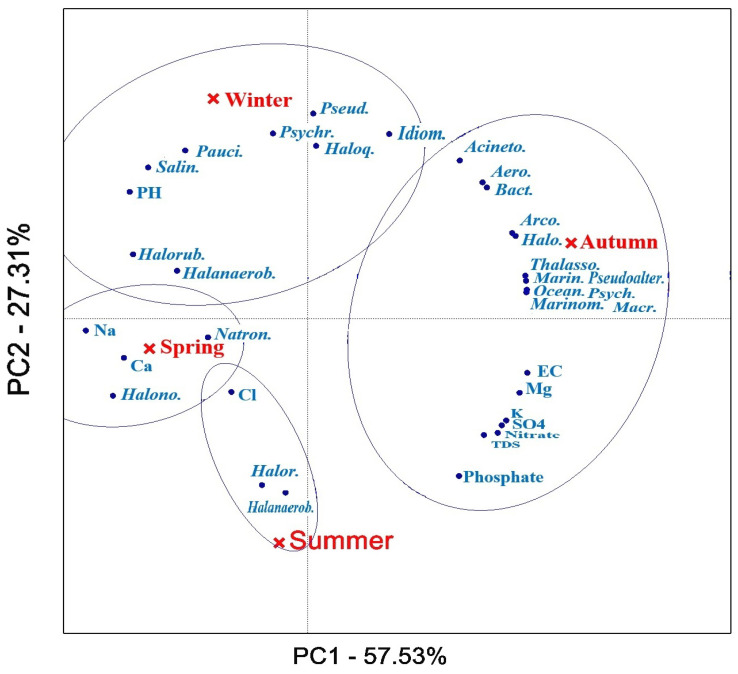
Principal component analysis of physico-chemical properties and bacterial dynamics across the four seasons (*Salin.*: *Salinibacter*; *Pauci.*: *Paucimonas*; *Halanaerob.*: *Halanaerobium*; *Halorub.*: *Halorubrum*; *Pseud.*: *Pseudomonas*; *Psychr.*: *Psychrobacter*; *Haloq.*: *Haloquadratum*; *Idiom.*: *Idiomarina*; *Acineto.*: *Acinetobacter*; *Aero.*: *Aeromonas*; *Bact.*: *Bacteroides*; *Arco.*: *Arcobacter*; *Halo.*: *Halomonas*; *Natron.*: *Natronomonas*; *Halono.*: *Halonotius*; *Halor.*: *Halorhabdus*; *Halanaerob.*: *Halanaerobacter*; *Thalasso.*: *Thalassospira*; *Marin.*: *Marinobacter*; *Ocean.*: *Oceanicaulis*; *Marinom.*: *Marinomonas*; *Psych.*: *Psychroflexus*; *Pseudoalter.*: *Pseudoalteromonas*; *Macr.*: *Macromonas*).

This PCA shows that both bacterial communities and environmental factors are significantly correlated with seasonal dynamics and microbial communities in Lake Urmia. The biplot associated with the PCA shows the explained variance for each component (Figure 5). Accordingly, the first two principal components (PCs) explained 57.53% and 27.31% of the variance, respectively. The first component explained most of the variance. Na^+^, Ca^2+^, *Natronomonas*, *Marinobacte*, *Thalassospira*, *Pseudoalteromonas* and *Oceanicaulis* have the greatest influence on the first component. *Pseudomonas*, *Halorhabdus*, *Paucimonas*, phosphate and pH have the greatest influence on the second component of the variations. The results of the PCA analysis show that the red ellipses group the data points based on seasonal clusters. Winter is associated with bacterial genera such as *Pseudomonas* and *Halorubrum* and correlates with pH and *Salinibacter*. Spring is associated with the variation of Na^+^, Ca^2+^ and *Natronomonas*. In addition, summer shows a correlation with phosphate and Mg^2+^, as well as with bacteria such as Halobacterium. Ref. [29] suggested that the high ion concentrations in hypersaline ecosystems may influence the micro-diversity profile of their highly abundant taxa. However, due to fluctuations in the volume and ionic composition of incoming water and evaporation and precipitation reactions, the chemistry of seawater is constantly changing. The water quality also varies seasonally and is determined by the inflows and the bathymetry of the lake [64].

## 4. Conclusions

The investigation of the physico-chemical properties of Lake Urmia revealed considerable seasonal fluctuations in the water quality parameters and their influence on the microbial communities. The seasonal variations in temperature, salinity, pH and nutrient concentrations in the lake significantly influence microbial diversity and community structure, highlighting the critical role of these environmental factors in shaping microbial dynamics. Analyzing the lake’s microbiome across different seasons showed that bacterial communities exhibit significant differences in diversity and composition. Alpha diversity metrics showed the highest genus richness in winter, while summer showed the lowest, indicating seasonal influences on microbial community structure. Dominance and rarity analyses show that winter and spring have higher core abundance and Gini index values, indicating a greater dominance of certain genera, while autumn and summer show a more balanced distribution. Analysis of beta diversity using the Bray–Curtis dissimilarity index emphasizes that winter and summer have the most distinct bacterial communities, reflecting the significant environmental changes between these seasons. The seasonal distribution of bacterial genera shows that some genera are present throughout the year, indicating their adaptability to different conditions, while others are season-specific, emphasizing the role of environmental factors in shaping microbial diversity. The presence of unique genera in each season is further evidence of the influence of seasonal changes on microbial community composition. Overall, understanding the seasonal variation in water chemistry and microbial communities is critical for effective ecosystem management and conservation efforts. The results highlight the complex interactions between environmental conditions and microbial communities and provide valuable insights into microbial adaptation and ecological dynamics in one of the world’s most unique and hypersaline aquatic ecosystems. Integrating these findings with environmental data can expand our knowledge of microbial ecology and support sustainable management practices for Lake Urmia and similar ecosystems.

## Figures and Tables

**Table 1:** Seasonal variations in the physico-chemical properties of Lake Urmia.

Parameter	Autumn	Winter	Spring	Summer
EC (ms/cm)	491.2 ± 10.2 a ^1^	226.5 ± 16.45 c	215 ± 3 c	348.2 ± 60.3 b
PH (mg/L)	7.08 ± 0.11 b	7.73 ± 0.12 a	7.6 ± 0.26 a	7.14 ± 0.16 b
TDS (mg/L)	410.4 ± 17.1 a	315.3 ± 4.8 b	306.5 ± 6.06 b	406.3 ± 90.3 a
Sodium (Na^+^) (mg/L)	58,918.9 ± 3710 c	72,252.9 ± 1641.9 ab	79,337.8 ± 1697.8 a	70,417.7 ± 7298.3 b
Calcium (Ca^2+^) (mg/L)	137.7 ± 48.4 a	179.03 ± 44.5 a	271.1 ± 94.0 a	185.5 ± 98.4 a
Magnesium (Mg^2+^) (mg/L)	34,717.7 ± 4166.1 a	14,245.3 ± 79.7 c	17,678.06 ± 1409.8 c	24,570.5 ± 5407.6 b
Potassium (K^+^) (mg/L)	6950.6 ± 507.5 a	3150.2 ± 180.4 c	3435.9 ± 381.2 c	5854.9 ± 836.7 b
Chloride (Cl^−^) (mg/L)	201,167.2 ± 5422.6 a	195,508.3 ± 16,485.6 a	214,775.2 ± 6400.1 a	200,871.1 ± 22,104.2 a
Sulfate (SO_4_^2−^) (mg/L)	42,179.4 ± 840.1 a	21,229.7 ± 1733.7 c	23,162.1 ± 1017.4 c	36,727.8 ± 4931.1 b
Phosphate (PO_4_^3−^) (mg/L)	0.26 ± 0.04 a	0.163 ± 0.05 b	0.193 ± 0.015 b	0.27 ± 0.03 a
Nitrate (NO_3_^−^) (mg/L)	67.1 ± 5.7 a	26.73 ± 3.5 b	32.43 ± 5.03 b	57.6 ± 13.9 a

^1^ Different letters indicate statistically significant differences within a row according to Duncan’s test (significant at *p* < 0.05). Values with the same letter indicate no statistically significant difference within a row (*p* > 0.05).

**Table 2:** Alpha diversity indices of bacterial communities in Lake Urmia across the four seasons.

Season	Observed Species	Shannon	Simpson	InvSimpson
Autumn	71	1.835828	0.7748228	4.440946
Winter	72	1.516104	0.7320779	3.732428
Spring	37	1.566810	0.7538326	4.062276
Summer	26	1.664539	0.7877350	4.711091

**Table 3:** Dominance and rarity analysis of bacterial communities in Lake Urmia across the four seasons.

	dbp	dmn	Absolute	Relative	Simpson	Core_Abundance	Gini
Autumn	0.323	0.616	38652	0.323	0.225	0.908	0.957
Winter	0.358	0.665	65596	0.358	0.268	0.989	0.968
Spring	0.359	0.604	60179	0.359	0.246	0.995	0.967
Summer	0.288	0.532	27542	0.288	0.212	0.994	0.963

**Table 4:** Analysis of core abundance of bacterial genera in Lake Urmia across the seasons.

	Camargo	Pielou	Simpson	Evar	Bulla
Autumn	0.068966	0.430675	0.062549	0.085036	0.140339
Winter	0.049963	0.354506	0.051839	0.091282	0.081367
Spring	0.176623	0.433909	0.109791	0.051493	0.143565
Summer	0.231554	0.510893	0.181196	0.04837	0.206026

**Table 5:** Bray–Curtis dissimilarity matrix between samples collected in different seasons.

	Autumn	Winter	Spring	Summer
Autumn	-	0.28351291	0.24116225	0.24343426
Winter	0.28351291	-	0.08281522	0.35045450
Spring	0.24116225	0.08281522	-	0.29444537|
Summer	0.24343426	0.35045450	0.29444537|	-

## Data Availability

Data are available upon reasonable request.
